# Supplementary material for: Signaling Switching from Hedgehog-GLI to MAPK Signaling Potentially Serves as a Compensatory Mechanism in Melanoma Cell Lines Resistant to GANT-61
Source: Biomedicines. 2023 May 3;11(5):1353. doi: 10.3390/biomedicines11051353 (PMC10216463; doi:10.3390/biomedicines11051353)
Supplement: Supplementary file 1 [file biomedicines-11-01353-s001.zip › biomedicines-2329375-supplementary.pdf]

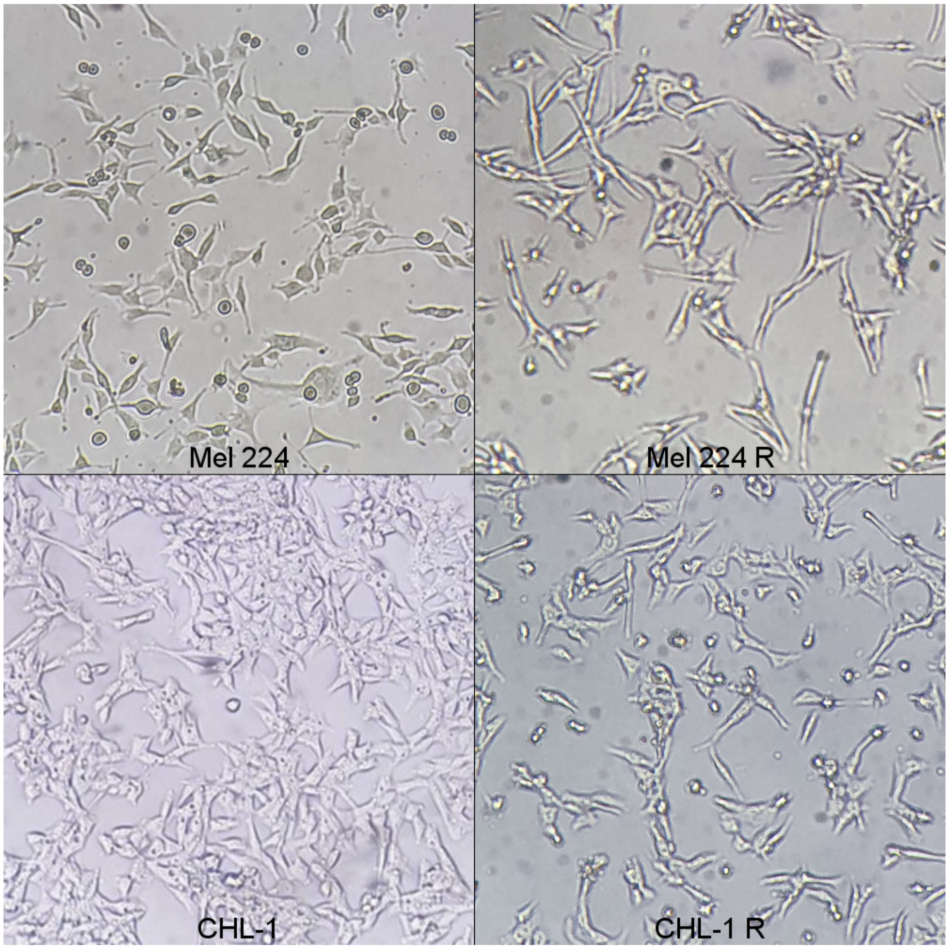

Figure S1. Morphology of established cell lines resistant to GANT-61.

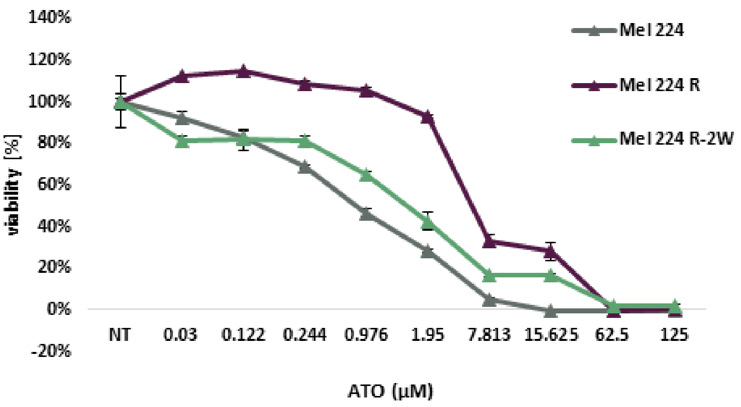

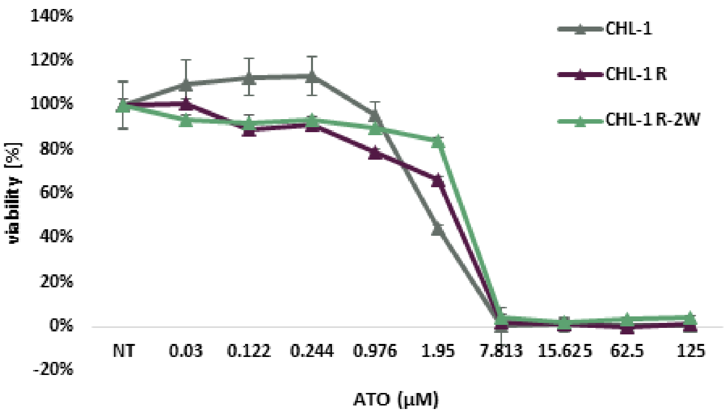

Figure S2. Cell viability examined with MTT after arsenic trioxide treatment in established cell lines resistant to GANT-61.

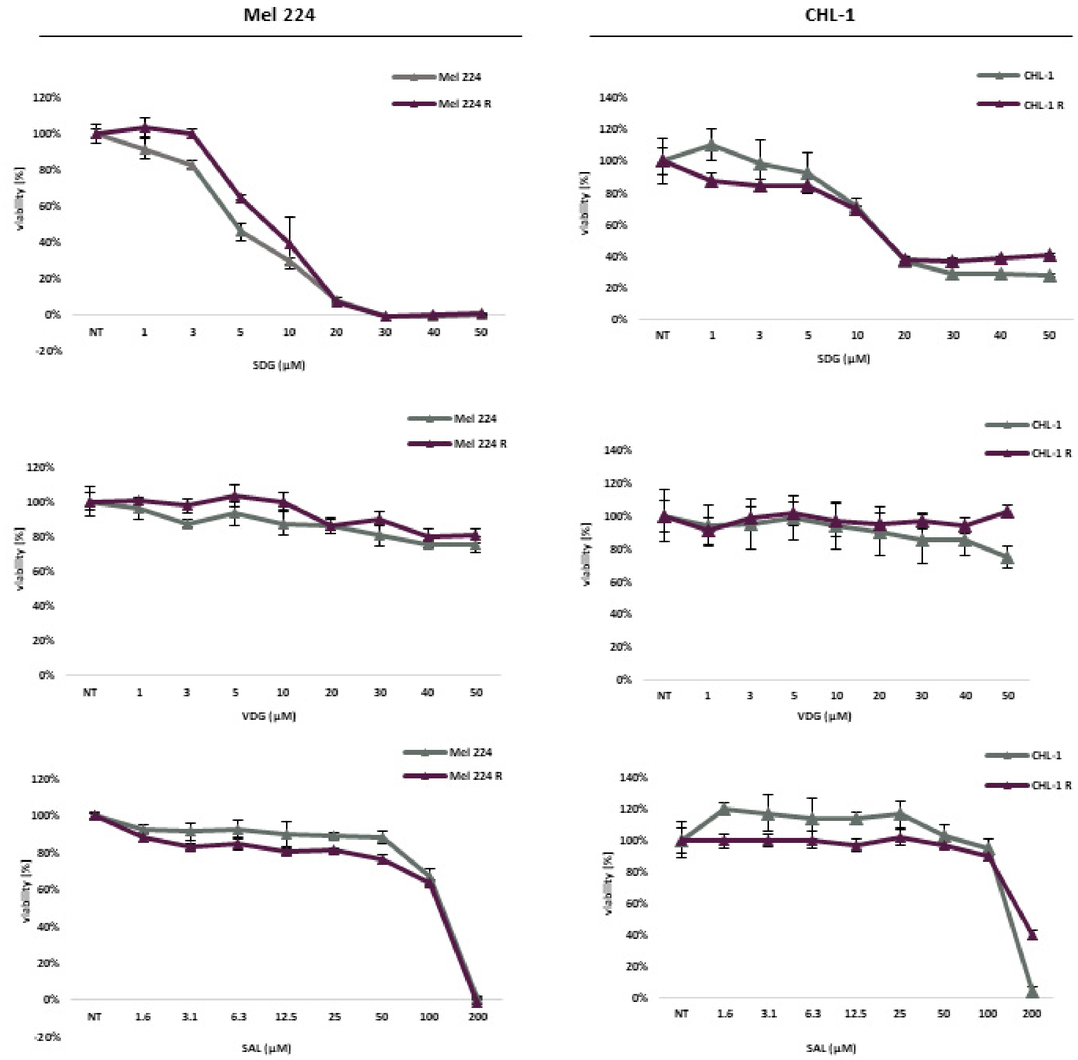

Figure S3. Cell viability examined with MTT after sonidegib (SDG), vismodegib (VDG) and salirasib (SAL) treatment in established cell lines resistant to GANT-61.

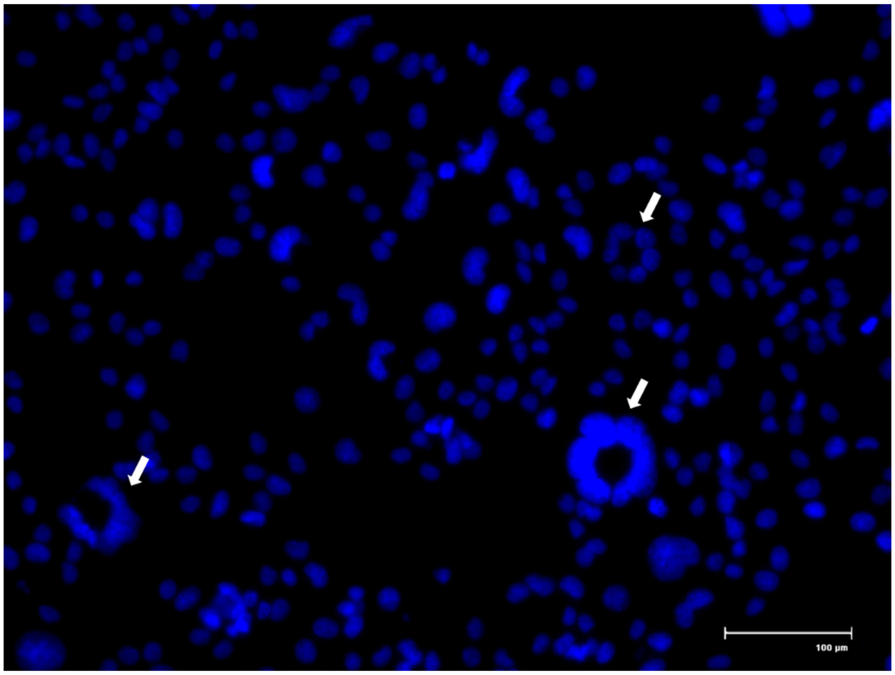

Figure S4. Morphology of multinuclated cells (MC) after staining with DAPI. In this figure, multinucleated cells identified in Mel 224 R were presented. Multinuclated cells are hihlighted with white arrows.
